# Sarcopenia in Type 2 Diabetes Mellitus: A Cross-Sectional Observational Study

**DOI:** 10.1155/2020/7841390

**Published:** 2020-10-29

**Authors:** L. M. Pechmann, T. H. Jonasson, V. S. Canossa, H. Trierweiler, G. Kisielewicz, R. R. Petterle, C. A. Moreira, V. Z. C. Borba

**Affiliations:** ^1^Internal Medicine and Endocrine Division (SEMPR), Universidade Federal do Paraná, Centro de Diabetes Curitiba, Curitiba, PR, Brazil; ^2^Endocrine Division, Hospital de Clínicas da Universidade Federal Do Paraná (SEMPR), Avenida Agostinho Leão Júnior, 285, Alto da Glória, Curitiba, PR 80030-110, Brazil; ^3^Universidade Federal do Paraná, Curitiba, PR, Brazil; ^4^Department of Integrative Medicine, Federal University of Paraná, Curitiba, PR, Brazil

## Abstract

**Background:**

The aim of this study was to compare the prevalence of low muscle mass and sarcopenia in patients with type 2 diabetes mellitus (T2DM) versus paired controls (control group, CG) and the association between sarcopenia and chronic diabetes complications.

**Methods:**

Men and women ≥50 years with T2DM (T2DM group, T2DMG) were recruited during routine outpatient visits. Total body densitometry and handgrip strength (HGS) were evaluated in the T2DMG and CG, while the T2DMG was also evaluated for the physical performance using the gait speed (GS) test. Sarcopenia was diagnosed according to the criteria of the Foundation for the National Institutes of Health Sarcopenia Project (FNIH).

**Results:**

The study included 177 individuals in the T2DMG and 146 in the CG. The mean HGS value was lower in the T2DMG (24.4 ± 10.3 kg) compared with the CG (30.9 ± 9.15 kg), *p* < 0.001, with low HGS in 46 (25.9%) and 10 (9%) in the T2DMG and CG, respectively (*p* < 0.001). The prevalence of sarcopenia defined according to the FNIH criteria was higher in the T2DMG 23 (12.9%) compared with the CG 8 (5.4%), *p* < 0.03. The presence of albuminuria increased the odds of sarcopenia (odds ratio (OR) 2.84, 95% confidence interval (CI) 1.07–7.68, *p*=0.04) and osteoporosis (OR 3.38, 95% CI 1.12–9.89, *p*=0.03), even in patients with mild to moderate nephropathy. The body composition analysis showed increased odds of sarcopenia with increased percentage of total fat (%TF) in women (OR 1.18, 95% CI, 1.03–1.43, *p*=0.03) and men (OR 1.31, 95% CI, 1.10–1.75, *p*=0.01).

**Conclusion:**

Patients with T2DM presenting with albuminuria, osteoporosis, and increased %TF were more likely to have sarcopenia. This finding emphasizes the need for patients with T2DM to be evaluated for sarcopenia to allow for early implementation of measures to prevent or treat this disorder.

## 1. Background

Type 2 diabetes mellitus (T2DM) is a highly prevalent chronic disease. The International Diabetes Federation estimated that 463 million individuals had diabetes mellitus in 2019, and that 90% of these individuals had T2DM [1]. After the third decade of life, muscle mass decreases at a yearly rate of up to 1% [2]. Sarcopenia occurs when this loss is excessive and accelerated, leading to muscle function deterioration and impairment [3]. Both sarcopenia and T2DM become more prevalent with aging and predispose patients to long-term complications, frailty, hospitalizations, and premature death. Sarcopenia occurs 3–16 times more frequently in patients with diabetes [4, 5].

In addition to its role in mobility, the skeletal muscle also plays an important role in metabolism and is crucial in glucose homeostasis. Chronic kidney disease (CKD), one of the microvascular complications of diabetes, is characterized by abnormalities in renal structure or function. Urinary albumin excretion is an early indicator of renal damage in patients with diabetes and a key factor in the development of diabetic nephropathy [6]. Albuminuria is also associated with increased abdominal obesity and visceral fat. In turn, visceral fat accumulation and secretion of cytokines by visceral adipose tissue—both typically seen in patients with T2DM—also contribute to glomerular sclerosis and renal function damage [7]. Albuminuria is a major risk factor for renal and cardiovascular events in T2DM, indicating the importance of early identification of this complication.

Sarcopenia occurring in early CKD stages is poorly characterized; still, this complication should be taken into account when albuminuria is detected. Albuminuria can serve an alert for both CKD and sarcopenia, especially in cases of unsuspected sarcopenia [8, 9]. Some studies have suggested that increased lean mass (LM) is a protective factor against renal dysfunction, since patients with greater LM tend to have lower albuminuria. Other pathophysiological changes that contribute to sarcopenia in CKD include increased inflammation, protein loss, reduced vitamin D synthesis, metabolic acidosis, and mitochondrial dysfunction due to muscle breakdown, leading to loss of strength and muscle mass and resulting in sarcopenia [7–9].

Based on the considerations above, the aim of this study was to compare the prevalence of low muscle mass and sarcopenia in patients with T2DM compared with controls, and the association of low muscle mass and sarcopenia with T2DM comorbidities and chronic complications, mainly nephropathy.

## 2. Methodology

### 2.1. Study Sample and Data Collection

This cross-sectional study evaluated a group of patients with T2DM (T2DM group, T2DMG) recruited by convenience during routine visits to the outpatient clinic of Serviço de Endocrinologia e Metabologia (SEMPR) of Hospital de Clínicas at Universidade Federal of Paraná between March and October 2017. We included men and women aged ≥50 years and with a diagnosis of T2DM for at least 1 year. We excluded patients with type 1 diabetes mellitus, those with a body mass index (BMI) >40 kg/m^2^ or <18 kg/m^2^, and individuals receiving treatment for chronic diseases and hormonal treatment or medications that affect muscle mass or could directly modify body composition (such as diuretics or corticosteroids). We also excluded patients taking nutritional supplements with high protein content, professional athletes, and individuals who were immobilized, disabled, or could not follow the evaluation protocol. The occurrence of diabetes complications (cardiovascular disease, retinopathy, peripheral neuropathy, and nephropathy) was retrieved from medical records and confirmed by the clinician in charge of the patient. The most recent biochemical evaluation including serum creatinine (10–15 days before the visit) and albuminuria (morning sample, 3–6 months before the visit) results were also collected from medical records [6].

The control group (CG) comprised healthy community-dwelling individuals identified in the SEMPR database. Individuals in this group were matched (using the frequency matching method) by age and sex with the participants in the T2DMG. Exclusion criteria in this group included the presence of chronic diseases under current treatment, a diagnosis of T2DM or glucose intolerance (as either impaired fasting glucose or impaired glucose tolerance), BMI > 40 kg/m^2^ or <18 kg/m^2^, use of nutritional supplements with high protein content, hormonal treatment, or use of medications that affect muscle mass or may directly modify body composition (such as diuretics and corticosteroids). Professional athletes and immobilized or disabled individuals were also excluded. The participants in the CG answered the same questionnaires and underwent the same evaluations as those in the T2DMG, with the exception of physical performance, which was not evaluated in this group. The dual-energy X-ray absorptiometry (DXA) equipment and the technician who performed this evaluation were the same in both groups.

### 2.2. Questionnaires

All patients and controls answered a structured questionnaire developed by the researchers to collect demographic and baseline characteristics, comorbidities, continuous medications in use, life habits (smoking, alcoholism, calcium consumption, and physical activity). The dietary habits of the participants were classified according to the Dietary Guidelines for the Brazilian population. The patients were considered to be healthy or unhealthy if they consumed in excess or less than six servings of fruits and vegetables per day, respectively. Based on the dietary recommendations, the daily intake of calcium was classified as sufficient when greater than 1200 mg [10].

### 2.3. Anthropometric Measures

Body weight was measured with the participants wearing light clothes and no shoes, using a scale with a measuring accuracy of 100 g and capacity of up to 150 kg (Indústrias Filizola SA, São Paulo, SP, Brazil). Height was measured with a Tonelli stadiometer (IN Tonelli—SA, Santa Catarina, Brazil) with 0.1 cm accuracy. Waist circumference was classified according to the International Diabetes Federation and National Cholesterol Education Program-Adult Treatment Panel III (ATP III) criteria. Values ≥102 cm and ≥88 cm in men and women, respectively, were considered increased [11, 12].

### 2.4. Body Composition and Bone Mineral Density Evaluation

Spine and hip bone mineral density (BMD) and body composition were analyzed by DXA in all participants using a Lunar Prodigy device (GE Medical Systems, Madison, WI, USA) equipped with the Encore software. BMD was analyzed following the International Society for Clinical Densitometry guidelines [13] and classified according to the World Health Organization (WHO) criteria [14]. All DXA evaluations were performed by a certified operator and analyzed by a certified radiologist. For body composition analysis, total fat (TF), percentage of TF (%TF), percentage of android fat (%AF), and total LM (TLM) were measured in the overall cohort and grouped by sex. We calculated the participants' appendicular LM (ALM) as the sum of the LM values obtained in the arms and legs, the ratio of ALM over BMI [15], and the relative skeletal muscle index (RSMI) as the ratio of ALM over the squared height [16]. Nine patients did not undergo body composition evaluation on the same day as the BMD scan and refused to return to the clinic for the evaluation.

### 2.5. Handgrip Strength

We measured the participants' HGS using a medical handgrip dynamometer (MG 4800, Charder Electronic Co., Ltd., Taichung City, Taiwan) according to the methodology recommended by Roberts et al. [17]. Each individual sat on a chair with armrests after removing rings, watches, and other objects from their hands and wrists. The arm to be evaluated was placed alongside the body with the elbow at a 90° angle, while the opposite arm rested on the thigh. The examiners were instructed to provide a verbal motivational stimulus to determine the individual's maximum strength at each measurement. Three measurements were obtained for each hand in an alternating manner, and the maximum strength was defined as the greatest of all six measurements. Weakness was defined according to the Foundation for the National Institutes of Health Sarcopenia Project (FNIH) cutoff point as a HGS < 26 kg in men and <16 kg in women [15].

### 2.6. Low Muscle Mass

Low muscle mass was diagnosed according to the cutoff values established by the FNIH criteria (ALM/BMI < 0.789 for men and <0.512 for women) [15].

### 2.7. Performance Assessment

Physical performance was tested after proper instructions in 93 participants with the T2DMG using the GS test. The participants were asked to stand still with their feet behind a starting line marked with tape and, after the examiner's command of “go,” were instructed to walk a 6-meter course at their usual pace and stop just past the finish line. Timing was started when the participant's first foot was placed on the starting line and stopped when the first foot crossed entirely the finish line. Three assessments were obtained from all participants in the T2DMG. Individuals with GS ≤ 0.8 m/s were classified as having low physical performance [15].

### 2.8. Sarcopenia Diagnosis

Sarcopenia was diagnosed according to the FNIH criteria based on the presence of low muscle mass associated with low HGS or physical performance by GS [15].

### 2.9. Biochemical Evaluation

We obtained the following biochemical evaluations: fasting plasma glucose (hexokinase/glucose-6-phosphate dehydrogenase method; normal range (NR) < 100 mg/dL; coefficient of variation (CV) ≤ 5%), serum calcium (Arsenazo III method; NR 8.5–10.2 mg/dL; CV ≤ 3%), serum creatinine (Jaffe method; NR 0.8–1.3 mg/dL; CV ≤ 6%), glycated hemoglobin (National Glycohemoglobin Standardization Program; NR < 5.8% (International Federation of Clinical Chemistry, 68 mmol/mol); CV ≤ 5%, and the mean value of the most recent three tests was considered for the analysis), serum total cholesterol (colorimetric analysis; NR < 200 mg/dL; CV ≤ 3%), serum low-density lipoprotein (LDL; Friedewald equation; NR 85–125 mg/dL; CV ≤ 3%), serum high-density lipoprotein (HDL; colorimetric enzyme method; NR > 40 mg/dL in men and >45 mg/dL in women; CV ≤ 4%), serum triglycerides (glycerol-phosphate oxidase method; NR < 150 mg/dL; CV ≤ 5%), albuminuria (turbidimetric assay, NR < 30 mg/g; CV ≤ 5%, calculated by the albumin/creatinine ratio in a morning urine sample and classified as present when ≥30 mg/g) [6], and serum parathyroid hormone (chemiluminescence method; NR 15–68 pg/mL; CV ≤ 7%). GFR was calculated using the Modification of Diet in Renal Disease (MDRD) formula. Patients were classified according to serum 25-hydroxyvitamin D levels as deficient when <20 ng/mL, insufficient when <21–29 ng/mL, and normal when ≥30 ng/mL (immunochemiluminescence, LIAISON, DiaSorin, Saluggia, Italy, CV ≤ 5%) [18].

### 2.10. Statistical Analysis

All statistical analyses were performed using the software *R* (*R* Foundation for Statistical Computing, Vienna, Austria). Data are presented as mean ± standard deviation (SD) or median (minimum and maximum) values or as absolute and relative frequencies, as appropriate. The Kolmogorov–Smirnov test was used to evaluate the normality of the distribution of the variables. Quantitative variables were compared with Student's *t-*test for independent samples or with the nonparametric Mann–Whitney test. Qualitative variables were compared using Fisher's exact test or the chi-square test. Univariate analysis was performed using a logistic regression model that included sarcopenia as the dependent variable. For each variable included in the model, we tested the null hypothesis that the probability of sarcopenia was equal to that of any variable (lack of association between the variable and sarcopenia) versus the alternative hypothesis of different probabilities. The significance (*p*) of the statistical tests and odds ratios (ORs) with 95% confidence intervals (CIs) were also calculated. Based on the results obtained in the univariate analysis, a multivariate analysis (logistic regression) adjusted for age and sex was performed to evaluate the odds of the development of sarcopenia, including all variables that emerged as significant in the univariate analysis. *p* values below 0.05 were considered statistically significant.

## 3. Results

**Figure 1:**
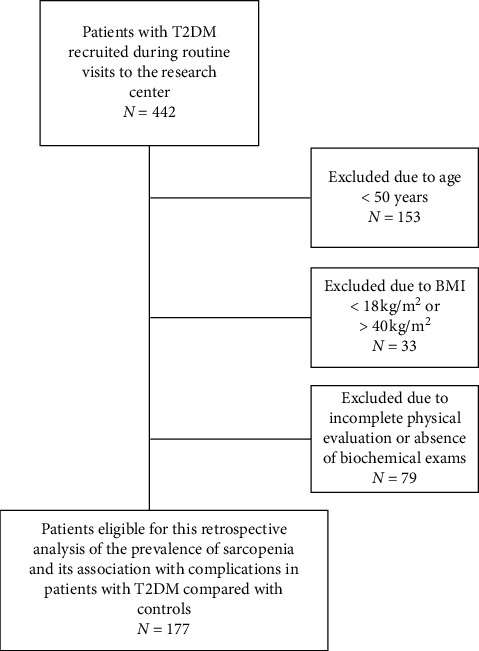
Study flow chart.

Of 442 patients with T2DM recruited, 265 did not meet the inclusion criteria (Figure 1); thus, the final sample comprised 177 patients (mean age 65.6 ± 8.6 years, 114 women). Of 300 controls identified in the database, 154 did not meet the inclusion criteria (of which age was the main reason), and the final sample in the CG comprised 146 individuals (mean age 65.0 ± 9.1 years, 80 women). In the T2DMG, the mean diabetes duration was 15.4 ± 8.2 years, the mean HbA1c level was 8.5 ± 5.7%, and 66.6% of the patients had a mean HbA1c above 7%, which is outside the target level recommended by the Brazilian Society of Diabetes [19] (Table 1).

### 3.1. Biochemical Analysis

The calculated GFR was below 60 mL/min (CKD stage 3A) in 52 (29.3%) patients in the T2DMG compared with individuals in the CG (*p* < 0.001). Albuminuria was detected in 49 (36.1%) patients in the T2DMG, including 16 (9.0%) patients who also presented GFR < 60 mL/min. Hypovitaminosis D and secondary hyperparathyroidism were observed in 80 (45.1%) and 46 (41.4%) patients, respectively, in the T2DMG (Table 1).

### 3.2. Body Composition and Bone Mineral Density

Compared with women in the CG, women in the T2DMG presented greater fat mass (FM), while men in this group had lower %AF compared with men in the CG. The mean values TLM, ALM, and RSMI were higher in the T2DMG compared with the CG in both sexes. After correction by BMI, ALM values were comparable in both groups. The %TF values (Table 2) and percentages of patients with low bone mass did not differ between groups. In the T2DMG, 72 (40.6%) patients had osteopenia and 27 (15.2%) had osteoporosis, whereas in the CG, the corresponding numbers were 53 (37.8%) and 23 (16.4%), respectively (*p*=0.71).

### 3.3. Evaluation of Muscle Strength and Physical Performance

The mean HGS value was lower in the T2DMG (24.4 ± 10.3 kg) compared with the CG (30.9 ± 9.15 kg; *p* < 0.001). According to the FNIH criteria, a low HGS was present in 46 (25.9%) participants in the T2DMG and in 10 (9%) of those in the CG (*p* < 0.001). In the T2DMG, the mean GS was 1.17 ± 0.27 m/s, and 14 (15.0%) patients in this group had a GS result below the FNIH cutoff value for low performance (<0.8 m/s).

### 3.4. Low Muscle Mass

**Figure 2:**
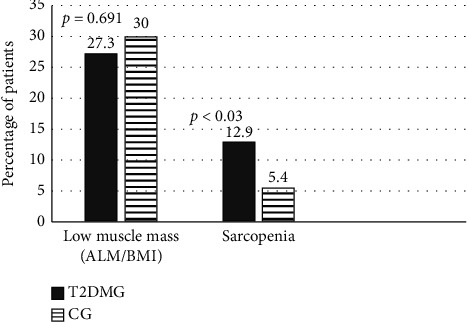
Prevalence of low muscle mass and sarcopenia in T2DMG and CG; abbreviations: T2DMG, T2DM group; CG, control group; ALM, appendicular muscle mass; BMI, body mass index; (*p*) statistically significant < 0.05.

The T2DMG had greater mean values of TLM and ALM. However, when ALM was corrected by BMI, the difference between groups was no longer significant (*p*=0.69) (Table 2, Figure 2).

### 3.5. Sarcopenia

The prevalence of sarcopenia defined according to the FNIH criteria was 12.9% (*n* = 23) in the T2DMG and 5.4% (*n* = 8) in the CG (*p* < 0.03). No difference in sex (*p*=0.307) or ethnicity (*p*=0.399) distribution was observed between groups.

### 3.6. Body Composition Analysis

Table S1 shows that women with sarcopenia had lower TLM, LM of the arms and legs, and ALM values compared with those without sarcopenia (*p* < 0.005 for all), but the difference in the ALM values were no longer present after ALM values were corrected by BMI (*p*=0.14). Individuals with sarcopenia had greater %TF and %AF values compared with those without sarcopenia.

When compared with T2DM patients without sarcopenia (T2DMGS−), T2DM patients with sarcopenia ( T2DMGS+) included more patients with an unhealthy diet, low performance, upper limb weakness, albuminuria, and wrist fractures (*p* < 0.05 for all). Among both men and women, sarcopenia was associated with %TF values (*p* < 0.05), but only in women was sarcopenia associated with AF (*p*=0.02) and TLM (*p*=0.03). There was a trend toward longer duration of T2DM and calcium plus vitamin D supplementation in the T2DMGS+ (*p*=0.06) Table 3. There was also a positive association between sarcopenia and presence of osteoporosis in the T2DMG (*p*=0.01) (Table 3).

Multivariate analysis was performed using sarcopenia as a dependent variable and including all variables that emerged as significant in the univariate analysis (unhealthy diet, osteoporosis, low performance, albuminuria, and wrist fractures) as independent variables. After binary logistic regression adjusted for age and sex, the odds of sarcopenia were increased by the presence of osteoporosis and albuminuria. When the body composition parameters (TLM, LM in arms and legs, %TF, and %AF) were included in the multivariate analysis conducted separately for each sex, %TF was associated with increased risk of sarcopenia in both sexes (Table 4).

## 4. Discussion

We found, in this study, a higher prevalence of sarcopenia in patients with T2DM compared with controls (12.9% versus 5.4%, respectively) when the presence of sarcopenia was established according to the FNIH criteria. This finding is aligned with results from other studies that showed rates of sarcopenia between 9.25% and 18% in patients with T2DM, depending on the population and method used to diagnose sarcopenia [20–27]. Even more relevant was the finding that the occurrence of albuminuria almost tripled the odds of sarcopenia; this has not been previously demonstrated in Brazilian studies of patients with T2DM but is consistent with results of other studies [4, 27, 28].

The study group comprised mostly white individuals, reflecting the ethnic profile of the population in southern Brazil, although the groups differed in relation to the distribution of ethnicity. We found no association between ethnicity and low muscle mass or sarcopenia. Patients with T2DM had a mean age of 65 years and were relatively younger than the participants in other studies. However, the inclusion of younger patients in our study is justified by the fact that sarcopenia occurs earlier in diabetes mellitus and is strongly related to increased frailty [20, 29–32].

The quality of the diet in the T2DMG was poorer than that in the CG but followed the dietary pattern of individuals with diabetes living in the southern region of Brazil [19]. Ethnic, cultural, and social habits typical of T2DMG populations may explain this finding. Even though protein intake was not evaluated in our cohorts, studies have shown that poor dietary habits, especially low protein consumption, are associated with an increased risk of sarcopenia in the elderly [32–34]. The profile of patients in our cohort included a history of long-standing T2DM, use of insulin, and poor glycemic control. Patients with sarcopenia had a tendency to have diabetes for a longer time (around 19 years) but had similar HbA1c levels as those without sarcopenia. Both these groups had mean HbA1c levels above the recommended goal outlined in the statement of the Sociedade Brasileira de Diabetes (SBD) but aligned with mean national levels reported in other Brazilian studies [35].

The patients in the T2DMG in our study had high rates of common diabetes comorbidities such as dyslipidemia and hypertension. Hypertension was almost three times higher in the T2DMG compared with the CG, which is aligned with Brazilian data showing a 2.4 times higher rate of hypertension in patients with T2DM [36]. Hypertension treatment with angiotensin-converting enzyme inhibitors could have prevented muscle loss in our study participants and may have interfered with the rates of sarcopenia, as these agents prevent declines in skeletal muscle mass and strength independent of their hemodynamic effects [37].

Many years before the diagnosis of T2DM, patients may develop insulin resistance (IR) often associated with central obesity. Both IR and central obesity have muscle effects, from the disease onset to advanced disease stages [38]. Patients with T2DM have shown altered expression of proteins involved in myogenesis, including myostatin, an inhibitor of skeletal muscle growth [32]. Both IR and hyperglycemia increase circulating free fatty acids leading to enhanced deposition of lipids and adipocytes within and around the muscle causing myosteatosis. This is a plausible cause for muscle strength decline in patients with T2DM and a possible explanation for the nonlinear relationship seen between decreased muscle mass and function/strength [34]. Our study showed that loss of muscle strength was more prevalent than decreased muscle mass in the T2DMG. Yoo et al. observed that muscle atrophy is more severe in type II muscle fibers and associated with greater strength loss in patients with T2DM [29]. Early changes in muscle quality (myosteatosis) and function in these patients justify the observed discrepancy between different criteria for assessing low muscle mass, suggesting that among patients with T2DM, ALM should be corrected by BMI in addition to the squared height, as indicated by the largest epidemiological, cross-validated study to date, the FNIH Sarcopenia Project [15].

Independent of sex, patients with T2DM and sarcopenia had higher %TF and %AF compared with those without sarcopenia. These differences may be explained by lower levels of physical activity and excessive caloric intake in patients with T2DM, suggesting that these unhealthy habits may contribute to changes in body composition. Almost 85% of the patients with T2DM and sarcopenia had upper limb weakness, and a possible explanation for this low muscle strength may be the presence of advanced glycation end products (AGEs), in which patients with T2DM accumulate more markedly in the skeletal muscle and are associated with lower HGS and GS in the elderly [20].

An important finding of our study was the association of sarcopenia with the presence of albuminuria. One-third of our sample had some impairment in renal function associated with albuminuria, and we found an approximately 2.8-fold greater chance of sarcopenia in the presence of albuminuria. The interplay between albuminuria and sarcopenia is not fully understood, but a possible explanation could be IR, which can cause glomerular hyperfiltration, endothelial dysfunction, increased vascular permeability, and albuminuria even in individuals without diabetes [5]. Besides, albuminuria is an early indicator of renal damage and cardiovascular disease and a key factor for the development of sarcopenia in patients with T2DM [5, 9, 28]. Studies have shown that worsening renal function is related to sarcopenia in patients with end-stage CKD, but only a few studies have assessed the association between sarcopenia and early-stage CKD [27, 8]. This is surprising considering that muscle wasting is a cardinal feature of CKD [39]. In the present study, renal function was assessed based on the GFR equation (MDRD). We found no significant GFR differences between patients in the T2DM with and without sarcopenia, possibly due to the small sample size, relatively homogeneous study population, and decreased muscle mass having influenced the estimated GFR, overestimating the actual renal function. The association found between sarcopenia and albuminuria justify an early evaluation of body composition and risk factors for sarcopenia in patients with T2DM with early and intermediate CKD, considering the high prevalence of both sarcopenia and CKD in these patients [8, 9, 27, 39].

Limitations of this study include the cross-sectional design, which does not allow for a causal evaluation, and the absence of information on daily protein intake and absolute albuminuria values. Finally, the participants in the CG were not evaluated with the GS test; this was justified by the inclusion in this group of healthy individuals who presented no risk factors for sarcopenia. The strengths of the study include the acquisition of body composition using DXA, the evaluation of performance and strength tests in patients with T2DM, and the fact that the profile of this cohort is aligned with that of the Brazilian population.

## 5. Conclusion

As populations age, the incidence of T2DM and sarcopenia tends to increase. Our study showed that patients with T2DM have a higher prevalence of sarcopenia than controls. The 2.8-fold increased odds of sarcopenia in the presence of albuminuria serve as an alert for the impact of sarcopenia in diabetes. Prospective studies are needed to confirm the causal relationship between these pathologies and to identify the link between sarcopenia and albuminuria.

## Figures and Tables

**Table 1:** Clinical and biochemical characteristics of patients with type 2 diabetes mellitus (T2DM) and controls.

	T2DMG (*n* = 177)	CG (*n* = 146)	*p* value
Age, years (mean ± SD)	65.6 ± 8.6	65.0 ± 9.1	0.520
Gender			0.070
Female	114 (64.4%)	80 (54.7%)	
Male	63 (35.5 %)	66 (45.2%)	
Ethnicity			
Caucasians	149 (84.2%)	139 (95.2%)	0.440
Mulatto/black	26 (14.6%)	5 (3.42%)	<0.010
BMI, kg/m^2^ (mean ± SD)	29.2 ± 4.89	26.2 ± 3.10	<0.010
Waist circumference, cm (mean ± SD)	99.7 ± 11.0	89.3 ± 10.0	<0.010
Menopause	111 (95.6%)	69 (86.2%)	0.090
Current smoking	12 (6.77%)	6 (4.10%)	0.720
Alcohol intake	9 (5.08%)	NA	NA
Calcium intake, mg/day (mean ± SD)	403 ± 3.5	651 ± 3.8	<0.010
Unhealthy diet	78 (44%)	43 (29.4%)	0.020
Comorbidities			
Dyslipidemia	154 (87.0%)	35 (27.7%)	<0.001
Hypertension	145 (82.0%)	39 (30.3%)	<0.010
Hypothyroidism	50 (28.2%)	16 (12.4%)	<0.001
Complications			
Any complications	107 (60.4%)	NA	
Cardiovascular disease	53 (29.9%)	NA	
Retinopathy	52 (29.3%)	NA	
Peripheral neuropathy	50 (28.2%)	NA	
Nephropathy	45.0 (25.4%)	NA	
Treatment of diabetes			
Metformin	153 (86.4%)	NA	
Insulin plus oral agent	98 (55.3 %)	NA	
Oral agent only	56 (31.5 %)	NA	
Insulin only	22 (12.4%)	NA	
Biochemical analysis			
Glucose, mg/dL (mean ± SD)	162 ± 68.4	93 ± 18.5	<0.001
Creatinine, mg/dL (mean ± SD)	1.18 ± 1.1	0.92 ± 0.23	<0.001
Glomerular filtration rate (MDRD), ml/min/1.73 m^2^ (mean ± SD)	71.0 ± 19.0	78.0 ± 15.0	<0.010
Calcium, mg/dL (mean ± SD)	9.41 ± 0.9	9.38 ± 0.47	<0.001
Total cholesterol, mg/dL (mean ± SD)	171.6 ± 50.2	NA	NA
Triglycerides, mg/dL (mean ± SD)	154.1 ± 103.4	NA	NA
LDL cholesterol mg/dL (mean ± SD)	96.8 ± 39.1	NA	NA
PTH, pg/mL (mean ± SD)	72.7 ± 50.5	NA	NA
Vitamin D, ng/mL (mean ± SD)	29.6 ± 13.3	NA	NA
Deficient (<20 ng/mL)	80 (45.1%)	NA	NA
Insufficient (21 a 29 ng/mL)	47 (26.6%)	NA	NA
Normal (≥30 ng/mL)	50 (28.2%)	NA	NA
HbA1c (NGSP) (mean ± SD)	8.5% ± 5.7	NA	NA
Albuminuria (>30 mg/g·cr)	49 (36.1%)	NA	NA

Unhealthy dietary habits, <6 servings of fruits and vegetables/daily; statistical significance, *p* < 0.05. The data are described as absolute and relative (%) frequency or mean ± standard deviation. Abbreviations: T2DMG, diabetes mellitus group; CG, control group; BMI, body mass index; PTH, parathyroid hormone; HbA1c, glycated hemoglobin; NGSP, national glycohemoglobin standardization program; MDRD, modification of diet in renal disease; mg/g Cr, milligram per gram of creatinine.

**Table 2:** Body composition (BC) stratified by sex.

	Women		Men			
BC parameters	T2DMG (*n* = 114)	CG (*n* = 80)	*p* value	T2DMG *(n* = 63)	CG (*n* = 66)	*p* value
TLM (kg)	40.3 ± 6.3	36.1 ± 4.9	<0.001	54.9 ± 5.4	49.5 ± 5.6	<0.001
LM arms (kg)	4.32 ± 1.2	3.88 ± 7.3	<0.001	7.19 ± 5.7	5.69 ± 8.8	<0.001
LM legs (kg)	13.0 ± 3.2	11.2 ± 1.8	<0.001	18.2 ± 3.4	15.8 ± 2.2	<0.001
%TF	39.7 ± 6.4	40.2 ± 6.2	0.350	30.0 ± 6.6	30.6 ± 5.9	0.580
FM (kg)	27.7 ± 8.7	25.2 ± 6.5	0.030	23.9 ± 8.4	22.6 ± 5.7	0.320
AF (%)	46.1 ± 7.4	45.7 ± 7.4	0.640	38.3 ± 8.4	41.5 ± 6.9	0.020
ALM/BMI	0.58 ± 0.72	0.60 ± 1.03	0.450	0.86 ± 0.13	0.84 ± 0.25	0.570

BC, body composition; TLM, total lean mass; LM, lean mass; ALM, appendicular lean mass; TF, total fat; BMI, body mass index; FM, fat mass; AF, android fat; T2DMG, diabetes mellitus group; CG, control group.

**Table 3:** Variables associated with the presence or absence of sarcopenia in participants in the type 2 diabetes mellitus group (T2DMG).

	T2DMGS+ (*n* = 23)	T2DMGS− (*n* = 154)	*p*
Age, years (mean ± SD)	68.8 ± 11.0	65.1 ± 8.2	0.12
BMI, kg/m^2^ (mean ± SD)	30.0 ± 4.7	29.1 ± 4.8	0.39
Abnormal waist circumference	14 (60.8%)	8 (35.7%)	0.54
T2DM duration, years (mean ± SD)	18.7 ± 9.9	14.9 ± 7.7	0.06
Current smoking	8 (35.7%)	15 (65.2%)	0.84
Calcium intake, mg/day (mean ± SD)	508.48	388.36	0.47
Unhealthy diet	15 (65.2%)	63 (41.9%)	0.01
Calcium and vitamin D supplementation	12 (52.1%)	50 (32.4%)	0.06
Multivitamin supplementation	4 (17.3%)	19 (82.6%)	0.08
Hypertension	18 (78.2%)	5 (21.7%)	0.62
Hypothyroidism	9 (39.1%)	14 (26.6%)	0.21
Dyslipidemia	18 (78.2%)	5 (88.3%)	0.18
Depression	3 (13.0%)	20 (86.9%)	0.70
Arthrosis	7 (30.4%)	16 (69.5%)	0.11
COPD	2 (8.69%)	21 (91.3%)	1.00
Myocardial infarction	4 (18.1%)	18 (1.29%)	0.50
Retinopathy	8 (35.7%)	15 (65.2%)	0.54
Albuminuria	11 (50%)	38 (26%)	0.02
Glomerular filtration rate (MDRD) ml/min/1.73 m^2^ (mean ± SD)	71.49 ± 24.74	68.40 ± 18.17	0.19
Vitamin D, ng/mL (mean ± SD)	27.6 ± 10.6	29.7 ± 13.4	0.51
HbA1c, % (mean ± SD)	7.9% ± 1.5	8.5% ± 6.1	0.41
Gait speed, m/s (mean ± SD)	0.79 ± 0.1	1.22 ± 0.3	<0.010
Osteoporosis (%)	8 (34%)	21 (14%)	0.01
Wrist fracture (%)	4(17%)	7 (4%)	0.03
HGS (kg)	16.02 ± 5.68	27.75 ± 10.27	<0.001
ALM	16.23 ± 5.04	20.92 ± 6.78	<0.001
ALM/BMI	0.54 ± 0.12	0.73 ± 0.23	<0.001

The information on associated diseases was retrospectively retrieved from medical records through the participants' medical history using the International Statistical Classification of Diseases and Related Health Problems (ICD). Abbreviations: BMI, body mass index; MDRD, modification of diet in renal disease; HbA1c, glycated hemoglobin; COPD, chronic obstructive pulmonary disease; HGS, handgrip strength; ALM, appendicular lean mass; BMI, body mass index; T2DMGS+, T2DM patients with sarcopenia and T2DMGS−, T2DM patients without sarcopenia.

**Table 4:** Odds of sarcopenia and associated variables in participants in the type 2 diabetes mellitus group (T2DMG).

Variables	Sarcopenia OR (95% CI)	*p* value
Osteoporosis		
No	Reference	0.03
Yes	3.38 (1.12–9.98)	
Albuminuria		
No	Reference	
Yes	2.84 (1.07–7.68)	0.04
Gender		0.82
Female	Reference	
Male	0.88 (0.28–2.56)	
Percentage of total fat		
Men	1.31 (1.10–1.75)	0.01
Women	1.18 (1.03–1.43)	0.03
Age	1.05 (0.99–1.11)	0.10

CI, confidence interval; OR, odds ratio.

## Data Availability

The data used to support the findings of this study are available from the corresponding author upon request.

## Supplementary Materials

Supplementary Materials Table S1: body composition among participants in the type 2 diabetes mellitus group (T2DMG) with and without sarcopenia.
