# Supplementary material for: Sarcopenia in Type 2 Diabetes Mellitus: A Cross-Sectional Observational Study
Source: Int J Endocrinol. 2020 Oct 29;2020:7841390. doi: 10.1155/2020/7841390 (PMC7644336; doi:10.1155/2020/7841390)
Supplement: Supplementary Materials — Table S1: body composition among participants in the type 2 diabetes mellitus group (T2DMG) with and without sarcopenia. [file 7841390.f1.doc]

**Supplementary Table 1** - Body composition among participants in the type 2 diabetes mellitus group (T2DMG) with and without sarcopenia

| **Total**  **(*n* =177)** | **MEN**  **(*n*=63)** | | | **WOMEN**  **(*n*=114)** | | |
| --- | --- | --- | --- | --- | --- | --- |
| **Variable** | **T2DMGS+**  **(*n* = 6)** | **T2DMGS-**  **(*n*=57)** | ***p* value** | **T2DMGS+**  **(*N* = 17)** | **T2DMGS- (*n*=97)** | ***p* value** |
| **TLM, kg** | 55.1 ± 6.0 | 54.9 ± 5.4 | 0.95 | 35.7 ± 6.4 | 41.2 ± 5.9 | 0.030 |
| **LM arms, kg** | 7.3 ±6.0 | 6.0 ± 7.3 | 0.40 | 3.4 ± 8.1 | 4.4 ± 12.0 | <0.001 |
| **LM legs, kg** | 16.9 ± 2.6 | 18.3 ± 3.6 | 0.43 | 10.4 ± 2.2 | 13.5 ± 3.1 | <0.001 |
| **%TF** | 35.9 ± 4.4 | 29.3 ± 6.5 | 0.01 | 43.6 ± 5.4 | 39.0 ± 6.2 | 0.010 |
| **AF, %** | 45.1 ± 5.6 | 37.6 ± 8.3 | 0.05 | 50.2 ± 6.2 | 45.4 ± 7.4 | 0.002 |
| **FM, kg** | 27.1 ± 4.9 | 23.5 ± 8.7 | 0.20 | 26.3 ± 10.4 | 27.9 ± 8.4 | 0.570 |
| **ALM** | 22.9±3.1 | 24.8±4.9 | 0.38 | 13.7±2.9 | 18.0±4.2 | <0.01 |
| **ALM/BMI** | 0.72±0.34 | 0.87±0.16 | 0.39 | 0.47±0.03 | 0.62±0.12 | 0.14 |

Abbreviations: T2DMGS+, diabetes mellitus group with sarcopenia; T2DMGS-, diabetes mellitus group without sarcopenia; %TF, percentage of total fat; AF, android fat; TLM, total lean mass; LM, lean mass; ALM, appendicular lean mass; FM, fat mass; BMI, body mass index; ALM/BMI. Sarcopenia was defined according to FNIH criteria (16).
